# Associations between the triglyceride–glucose index and the risk of heart failure in patients undergoing maintenance hemodialysis: a retrospective cohort study

**DOI:** 10.3389/fendo.2025.1544591

**Published:** 2025-04-03

**Authors:** Qiuyue Zhang, Kai Zhou, Yuchen Li, Wen Dong, Yimiao Sun, Hui Wu, Xiaonan Qiu, Zhiyuan Liu, Ying Zhang

**Affiliations:** ^1^ The Affliated Hospital of Xuzhou Medical University, Xuzhou, Jiangsu, China; ^2^ Sihong People’s Hospital, Suqian, Jiangsu, China

**Keywords:** triglyceride-glucose index, heart failure, maintenance hemodialysis (MHD), insulin resistance, HF-related hospitalizations

## Abstract

**Background:**

Although the triglyceride-glucose (TyG) index levels have been shown to be a reliable predictor of major adverse cardiovascular events (MACE), few studies have investigated their association with heart failure (HF), especially in patients on dialysis. We therefore aimed to investigate the relationship between the TyG index and the incidence of HF in patients undergoing maintenance hemodialysis (MHD).

**Methods:**

A total of 183 participants who underwent MHD in the Blood Purification Center of the Affiliated Hospital of Xuzhou Medical University from September 2008 to October 2023 were included and followed up until March 2024. The TyG index was calculated as ln [fasting triglycerides (mg/dL) × fasting blood glucose (mg/dL)/2]. Participants were divided into two different groups according to the TyG index. The primary endpoint of this study was newly diagnosed HF events during the follow-up period. Cox proportional hazard models were used to examine the association between the TyG index and the risk of incident HF. To assess the dose-response relationship between TyG index and risk of HF, restricted cubic spline analysis was used.

**Results:**

Among the 183 participants, there were 61 incident cases of HF during a median follow-up period of 57 months. In comparison to the group with a lower TyG index, participants with a higher TyG index had a higher risk of HF (HR=2.590, 95%CI=1.490-4.500), regardless of whether a variety of potential confounders were adjusted. The association between TyG index and HF (P for non-linearity > 0.05) was confirmed by restricted cubic spline analysis.

**Conclusion:**

The TyG index was positively associated with the risk of incident HF in patients undergoing MHD, which indicates that the TyG index might be useful to identify people at high-risk for developing HF.

## Introduction

Heart failure(HF) represents a prevalent and significant complication in individuals with end-stage renal disease (ESRD), posing a grave threat to their quality of life and prognosis (1, 2). The incidence of HF among patients undergoing maintenance hemodialysis (MHD) has been documented to reach as high as 42.9%. Notably, the elevated incidence of HF in MHD patients cannot be entirely attributed to traditional risk factors. Rather, non-traditional risk factors, including renal insufficiency, chronic sodium and water retention, disturbances in calcium and phosphorus metabolism, uremic toxins, and arteriovenous fistulas, also contribute substantially to abnormalities in cardiac structure or function in this patient cohort (3, 4). Consequently, in-depth research into the risk factors for HF and identifying potential therapeutic targets in MHD patients holds crucial clinical significance.

Insulin resistance(IR) is a common risk factor for chronic kidney disease and heart failure (5, 6). While the hyperinsulinemic-euglycemic clamp technique is regarded as the closest to the gold standard for diagnosing IR. However, due to limitations such as complex operational procedures and high detection costs, the method is not suitable for clinical promotion and large-scale clinical studies. Triglycerides-glucose (TyG) index, used for assessment of IR, appears to be a valuable indicator, built mainly on the levels determined by fasting blood glucose (FBG) and triglycerides (TG) (7). Multiple clinical studies have indicated that the TyG index can effectively reflect the level of IR, which is measured through the hyperinsulinemic-euglycemia clamp technique (8). Furthermore, several studies have revealed associations between the TyG index and coronary artery calcification, as well as cardiovascular adverse events (9, 10). But, especially, whether the incidence of HF is affected by the TyG index has been insufficiently explored in all studies (11–13), which always excludes MHD patients.

With the decline in renal function, significant changes occur in lipid and glucose metabolism (14, 15). Research has revealed that perturbations in glucose and insulin homeostasis are already present in the early stages of CKD and intensify as renal function declines (16). In patients with ESRD, especially those requiring dialysis, there is even a phenomenon known as “reverse epidemiology” (17). With the heterodox lipid and sugar metabolism in the dialysis patients, the application of research results from people with normal renal function to the patients on dialysis may lead to the difficulty of buy-in. Therefore, in this study, we aimed to explore the relationship between the TyG index and the incidence of HF in the specific population of patients undergoing MHD.

## Materials and methods

### Study design and participants

**Figure 1:**
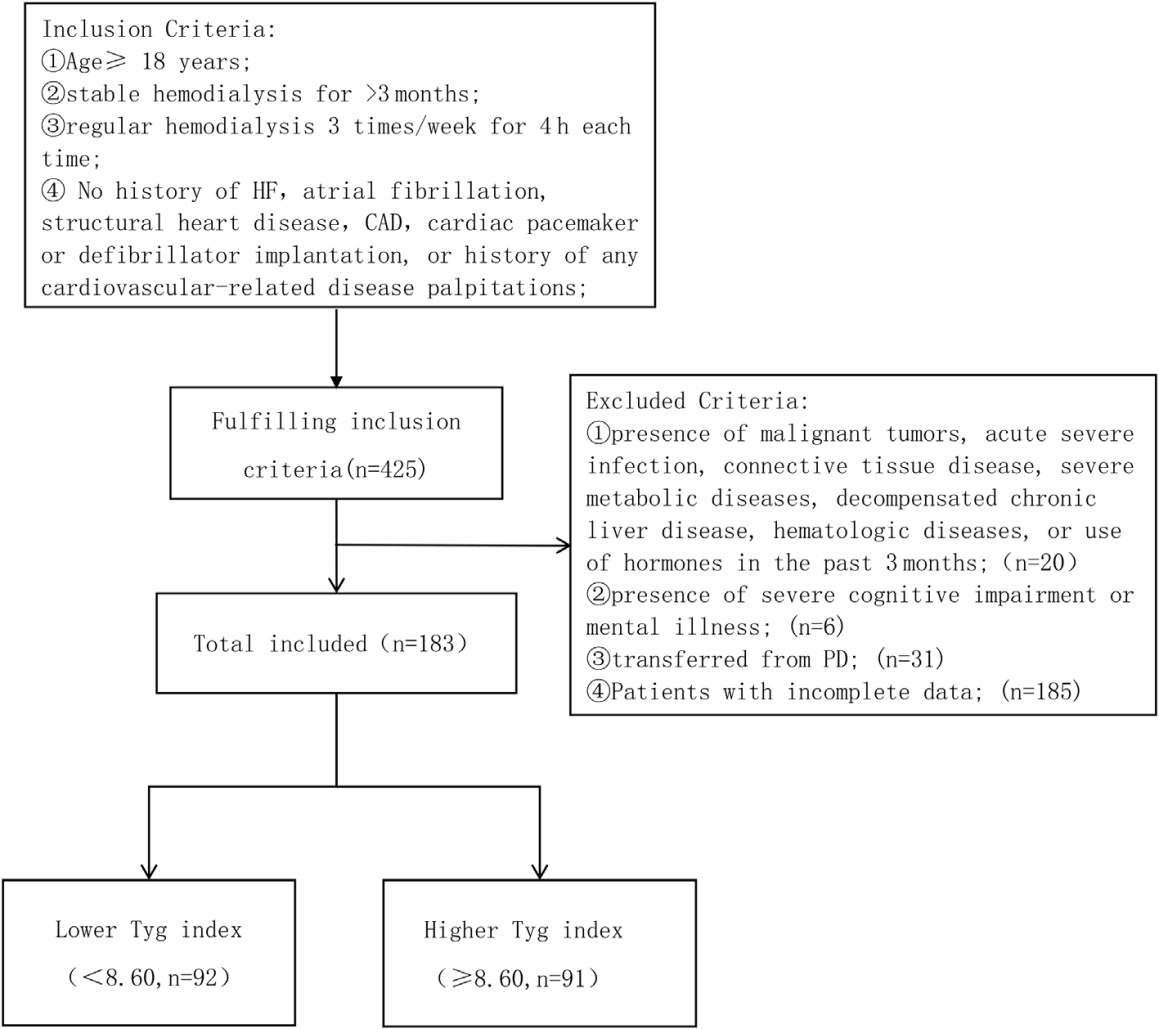
Enrollment flowchart for this study. TyG, triglyceride-glucose; HF, heart failure; CAD, Coronary Artery Disease.

This is a single-center retrospective study. From September 2008 and October 2023, patients who were undergoing maintenance hemodialysis at the Hemodialysis Center of Xuzhou Medical University were selected as study participants. The inclusion criteria are specified as follows: ①Age≥ 18 years ②stable hemodialysis for >3 months; ③regular hemodialysis 3 times/week for 4 h each time; ④ No history of HF, atrial fibrillation, structural heart disease, CAD, cardiac pacemaker or defibrillator implantation, or history of any cardiovascular-related disease; ①presence of malignant tumors, acute severe infection, connective tissue disease, severe metabolic diseases, decompensated chronic liver disease, hematologic diseases, or use of hormones in the past 3 months;(n=20) ②presence of severe cognitive impairment or mental illness; (n=6) ③transferred from PD; (n=31); ④Patients with incomplete data; (n=185) (**Figure 1**).

This study has been reviewed and approved by the Ethics Committee of the Affiliated Hospital of Xuzhou Medical University (ethics number: XYFY2024-KL495-01). Due to the retrospective nature of this study, informed consent was not required.

### Clinical data

The following demographic and clinical characteristic data were systematically collected: age, gender, BMI, history of hypertension, history of diabetes, history of cerebral infarction, primary underlying diseases, long-term medication history, heart rate, blood pressure, and dialysis vintage. Laboratory tests included measurements of hemoglobin, blood calcium, blood phosphorus, urea, blood creatinine, albumin, high-density lipoprotein, low-density lipoprotein, triglycerides, and fasting blood glucose. All laboratory test results were obtained from fasting blood samples collected before dialysis. Additionally, we calculated the triglyceride-glucose index (TyG index) using the formula: TyG index = ln [fasting TG (mg/dL) × fasting glucose (mg/dL)/2].

### Outcomes and follow-up

After enrollment, all maintenance hemodialysis (MHD) patients were followed up from the completion of baseline data collection until March 31, 2024. The primary endpoint was heart failure, diagnosed according to the criteria outlined in the 2021 European Society of Cardiology Guidelines for the Management of Heart Failure (18). Secondary endpoints included all-cause death and HF-related rehospitalization.

### Statistical analysis

The statistical analyses were carried out through the firm construction of SPSS version 25.0 and R software (version 4.4.1) programs. The Receiver Operating Characteristic (ROC) curve was employed for finding a cutoff and a relative level of a TyG Index, where an individual is considered either as a high TyG Index or a low TyG Index. All continuous variables were tested for normality. Variables that meet the conditions of a normal distribution were described as mean ± standard deviation, and the t-test was used to analyze the differences between the groups. Not normally distributed and continuous variables were represented as medians (25th, 75th percentile). They were checked for group differences through the use of nonparametric testing. Categorical variables were presented as frequencies and percentages, with intergroup comparisons conducted using the chi-squared test or Fisher’s exact test. KM survival analysis was used, in addition, to calculate the HF-free probability of survival amongst different TyG index groups. To investigate differences between the categories, the log-rank test was used. Univariate and multivariate Cox proportional hazards models followed to determine the effect of the TyG and the other variables on the clinical outcomes. The level of association between the outcome variables and the TyG Index was measured using hazard ratio (HR), and 95% confidence intervals (CIs) were computed. Besides, we applied the restricted cubic spline model to investigate the dose-response relationship between the TyG index and the risk probability of heart failure, where the number of knots was set to four. Subgroup analyses stratified by age, gender, quality of diabetes history, as well as BMI were studied, and the exploratory relations were presented using forest plots.

## Results

### Baseline characteristics of participants

**Figure 2:**
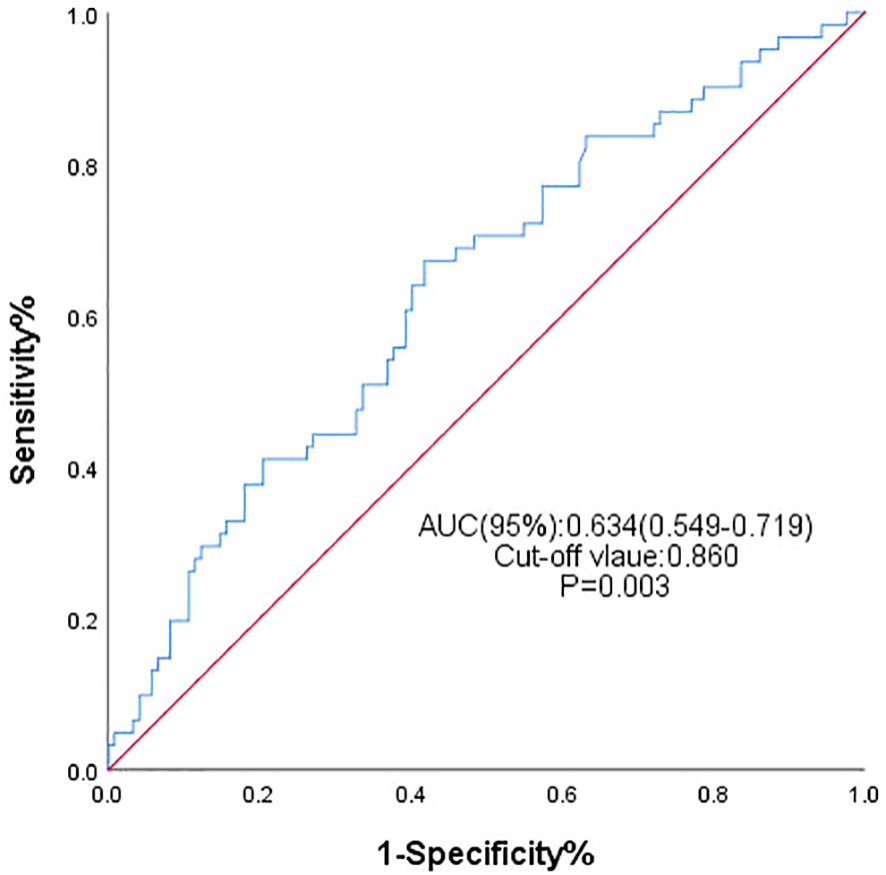
ROC curve of TyG index in predicting incident heart failure in MHD patients.

The study ultimately enrolled 183 MHD patients with a mean age of 54 years. Among them, 128 (69.9%) were male, and hypertension (79.3%) was the most common comorbidity. At the end of follow-up, a total of 61 patients developed HF. Among them, 49 subjects (80.33%) were diagnosed with HF with preserved ejection fraction (HFpEF), 1 subject (1.64%) was diagnosed with HF with reduced ejection fraction, and 11 subject (18.03%) was diagnosed with HF with mildly reduced ejection fraction. By plotting the ROC curve (**Figure 2**) and calculating the Youden index, the area under the curve was 0.634 (95% CI: 0.549–0.719, P=0.003). Grouping was performed based on the optimal cutoff value of the TyG index at 8.60, and **Table 1** displays the baseline clinical and laboratory characteristics of the two patient groups. Blood glucose, triglycerides, total cholesterol, and low-density lipoprotein cholesterol measurements in serum were all significantly higher in patients with the higher TyG index level, while high-density lipoprotein cholesterol was significantly lower in these patients. The main finding of this study was that the two groups had statistically significant differences in terms of the incidence of heart failure, with the higher TyG index group exhibiting a 44.6% incidence, which was higher than that of the other group, as shown in **Table 1**. Simultaneously, we observed that the incidence of HF-related rehospitalization in the group with a higher TyG index was 20.7%, which was also higher than that of the other group. There was no significant difference in all-cause mortality between the two groups.

**Table 1:** Comparison of baseline clinical characteristics between the two groups.

Characteristic	TyG index<8.60	TyG index≥8.60	t/z/χ²	*P*
Glucose, mmol/L	4.81 (4.43, 5.20)	5.58 (4.97,6.23)	-6.032	0
TG, mmol/L	1.00 (0.77,1.19)	1.92 (1.56,2.51)	-10.778	0
HF	20 (20%)	41 (44.6%)	10.503	0.001
Male (%)	25 (27.5%)	30 (32.6%)	0.574	0.449
Age, years	54.47 ± 13.66	53.62 ± 14.05	0.454	0.65
Duration of dialysis, mo	52.00 (28.00,83.00)	62.50 (23.25,89.25)	-0.522	0.602
BMI	23.66 (20.28,26.35)	23.41 (21.23,26.02)	-0.078	0.938
Heart rate, beats/min	79.00 (74.00,84.00)	78.00 (74.00,85.75)	-0.257	0.797
SBP, mmHg	150.50 ± 19.94	150.14 ± 23.99	0.076	0.94
DBP, mmHg	86.00 (80.00,100.00)	84.50 (78.00,97.75)	-1.176	0.239
Medical history, No. (%)				
Hypertension	72 (79.10%)	74 (80.40%)	0.049	0.825
Diabetes mellitus	17 (18.70%)	26 (28.3%)	2.335	0.126
Stroke	6 (6.60%)	12 (13.0%)	2.146	0.143
Diabetic nephropathy	12 (13.20%)	19 (20.70%)	1.812	0.178
Discharge medications, No. (%)				
Insulin therapy	9 (9.9%)	16 (17.4%)	2.182	0.140
ACE inhibitor or ARB	24 (26.40%)	28 (30.4%)	0.371	0.542
Beta-blocker	28 (30.80%)	40 (43.50%)	3.164	0.075
Erythropoietin	45 (49.5%)	37 (40.2%)	1.577	0.209
vitamin D	15 (16.50%)	19 (20.70%)	0.526	0.468
Hemoglobin, g/L	83.00 (73.00,91.00)	83.00 (69.00,97.75)	-0.267	0.79
TC, mmol/L	3.93 (3.33,4.65)	4.74 (3.99,5.75)	-5.25	0.000
HDL-C, mmol/L	1.11 (0.95,1.40)	1.00 (0.77,1.11)	-3.734	0.000
LDL-C, mmol/L	2.29 (1.75,2.66)	2.49 (2.01,3.24)	-2.893	0.004
Albumin, g/l	31.16 ± 5.48	37.53 ± 7.31	1.661	0.099
Ca (mmol/L)	2.05 (1.83,2.14)	2.00 (1.77,2.15)	-0.687	0.492
P (mmol/L)	1.97 (1.65,2.26)	2.08 (1.73,2.46)	-1.734	0.083
Serum urea, mmol/l	32.52 (25.96,41.00)	33.25 (26.70,44.38)	-1.073	0.283
Serum creatinine, mg/dl	809.00 (690.00,1051.00)	883.00 (739.00,1124.25)	-0.766	0.444
HF-related rehospitalization	8 (8.8%)	19 (20.7%)	5.117	0.024
All-cause death	13 (14.3%)	10 (10.9%)	0.486	0.486

BMI, Body mass index; ACE, angiotensin-converting enzyme; HF, heart failure; ARB, angiotensin receptor blocker; DBP, diastolic blood pressure; HDL-C, high-density lipoprotein cholesterol; LDL-C, low-density lipoprotein cholesterol; SBP, systolic blood pressure; TC, total cholesterol; TG, triglycerides; TyG, triglyceride-glucose; *Ca*, Calcium; *P*, Phosphorus.

### Association between TyG index and the risk of incident HF

**Figure 3:**
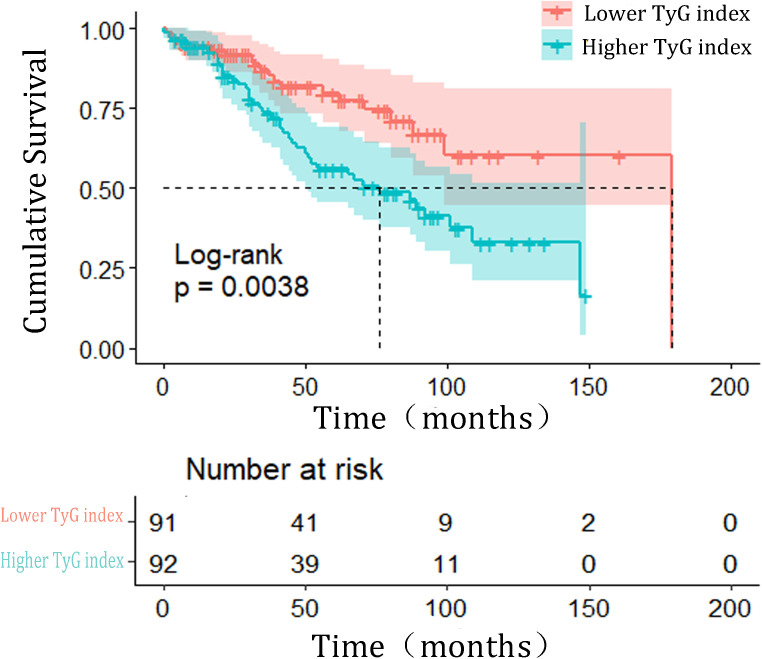
Kaplan–Meier estimated the incidence of HF events based on triglyceride-glucose index grouping. HF heart failure; TyG, triglyceride-glucose index.

**Figure 4:**
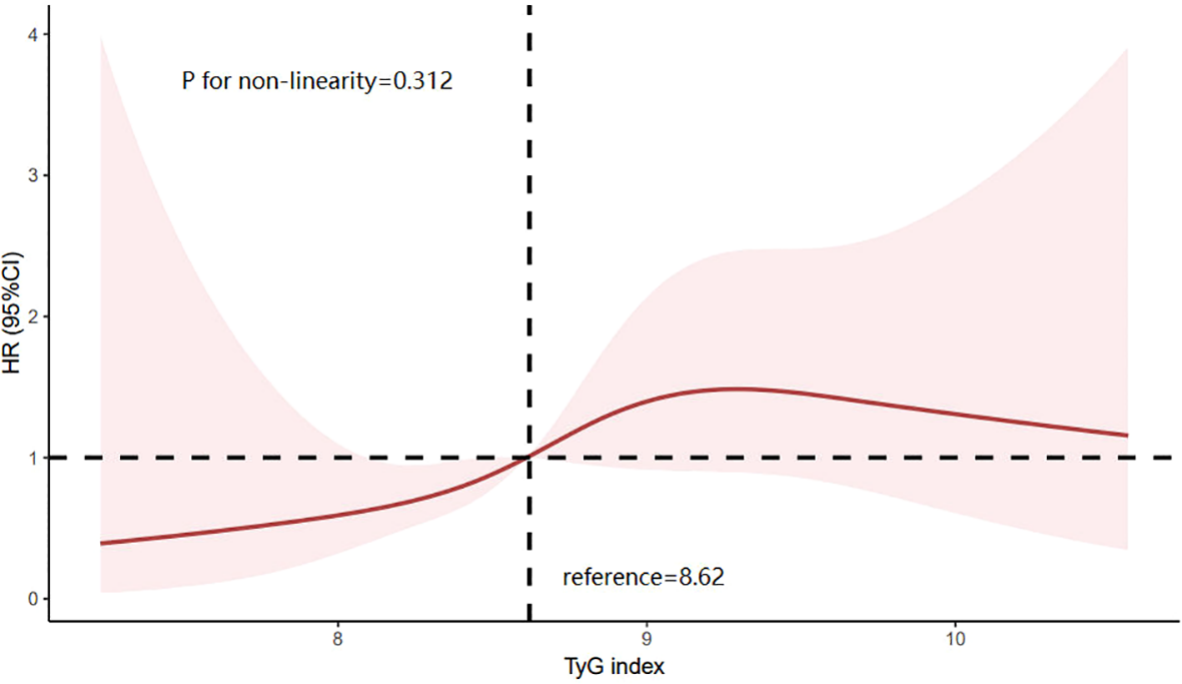
Limiting cubic spline of the relationship between triglyceride glucose index and the risk of incident HF. HR is indicated by a solid red line and 95% CI is indicated by a shaded area. CI, confidence interval; TyG index, triglyceride glucose index.

The Kaplan–Meier (KM) plot depicted in **Figure 3** illustrates that as compared to the lower TyG index group, the upper one correlates with a much higher risk of HF incidence (log-rank test P = 0.004). The TyG index is associated with higher values of hazard ratios for the incidence of HF in the population in question, as shown in **Table 2**. According to the univariate model (**Table 2**), it was diagnosed that diabetes, primary disease diabetic nephropathy, and higher BMI levels were predisposing factors for the onset of HF. Furthermore, the risk of incident HF was significantly associated with the TyG index (HR = 2.188, 95% CI 1.270–3.772, P = 0.005). The linear trend was similar when controlling for the potential variables (HR = 2.590, 95% CI 1.491–4.492, P = 0.001). Besides, exploration of the restricted cubic spline in relation to the TyG index revealed a consistent linear dose-response dependence upon the incident HF (P value nonlinearity > 0.05) (**Figure 4**).

**Table 2:** Univariate Cox regression model and Multivariate Cox regression model for the correlation between triglycerides-glucose index and the risk of incident heart failure.

Characteristic	Univariate analysis			Multivariate analysis		
	HR	95%CI	*P*	HR	95%CI	*P* ^a^
TyG index	2.188	1.270-3.772	0.005	2.59	1.490-4.500	0.001
Male	0.87	0.500-1.514	0.623	1.017	0.575-1.797	0.955
Age	1.012	0.993-1.031	0.223	1.011	0.989-1.032	0.339
BMI	1.057	1.000-1.116	0.048	1.068	1.002-1.139	0.044
Duration of dialysis	0.974	0.963-0.985	0.000	0.972	0.960-0.983	0
Diabetes mellitus	1.751	1.005-3.409	0.048	0.768	0.231-2.553	0.667
Stroke	1.930	0.912-4.087	0.086			
Diabetic nephropathy	2.178	1.207-3.933	0.01	2.212	0.634-7.714	0.213
Insulin therapy	1.879	0.992-3.561	0.053			
ACE inhibitor or ARB	1.624	0.955-2.764	0.074			
Beta-blocker	1.636	0.975-2.744	0.062			
DBP	0.987	0.971-1.003	0.099			
LDL-C,	1.222	0.987-1.513	0.066			
Serum creatinine,	0.999	0.999-1.000	0.05			
Glucose	1.09	0.998-1.191	0.055			

^a^Adjusted for covariates that were statistically significant in the univariate Cox regression model, including Diabetes mellitus, Duration of dialysis, BMI, TyG index. Clinically relevant risk factors were also adjusted in multivariate model, including age, male, BMI, Diabetes mellitus.

BMI, Body mass index; ACE, angiotensin-converting enzyme; ARB, angiotensin receptor blocker; DBP, diastolic blood pressure; HDL-C, high-density lipoprotein cholesterol; LDL-C, low-density lipoprotein cholesterol; TyG, triglyceride-glucose.

To assess the reliability of our primary findings, we conducted further analysis on 80 patients with baseline echocardiographic data. The Kaplan-Meier (KM) curve demonstrated that patients in the group with a higher TyG index had a significantly higher risk of HF (**Supplementary Figure 1**). Univariate Cox regression analysis revealed that higher levels of triglycerides (TG) and TyG index, a history use of beta-blocker were susceptible factors for the onset of heart failure, whereas higher levels of high-density lipoprotein cholesterol (HDL-C), a higher left ventricular ejection fraction (LVEF), and longer duration of dialysis were protective factors against heart failure. Multivariable Cox regression analysis showed a significant correlation between an elevated TyG index and an increased risk of HF (**Supplementary Table 2**). The area under the ROC curve was 0.704 (95% CI: 0.559 to 0.850, P = 0.012) (**Supplementary Figure 2**).

### Association between TyG Index and HF Rehospitalization and Mortality

In **Supplementary Table 3**, we analyzed the association between the TyG index and the risk of HF-related rehospitalization in 183 patients. In the multivariate Cox regression analysis of risk factors for HF-related rehospitalization, the TyG index (hazard ratio (HR) = 2.752, 95% confidence interval (CI) = 1.162-6.517, P = 0.021), BMI (HR = 1.127, 95% CI = 1.025-1.240, P = 0.013), and duration of dialysis (HR = 0.996, 95% CI = 0.946-0.985, P = 0.001) were found to be associated with HF-related rehospitalization. Additionally, in **Table 1**, we found no significant difference in all-cause mortality between the two groups.

### Subgroup analysis

**Figure 5:**
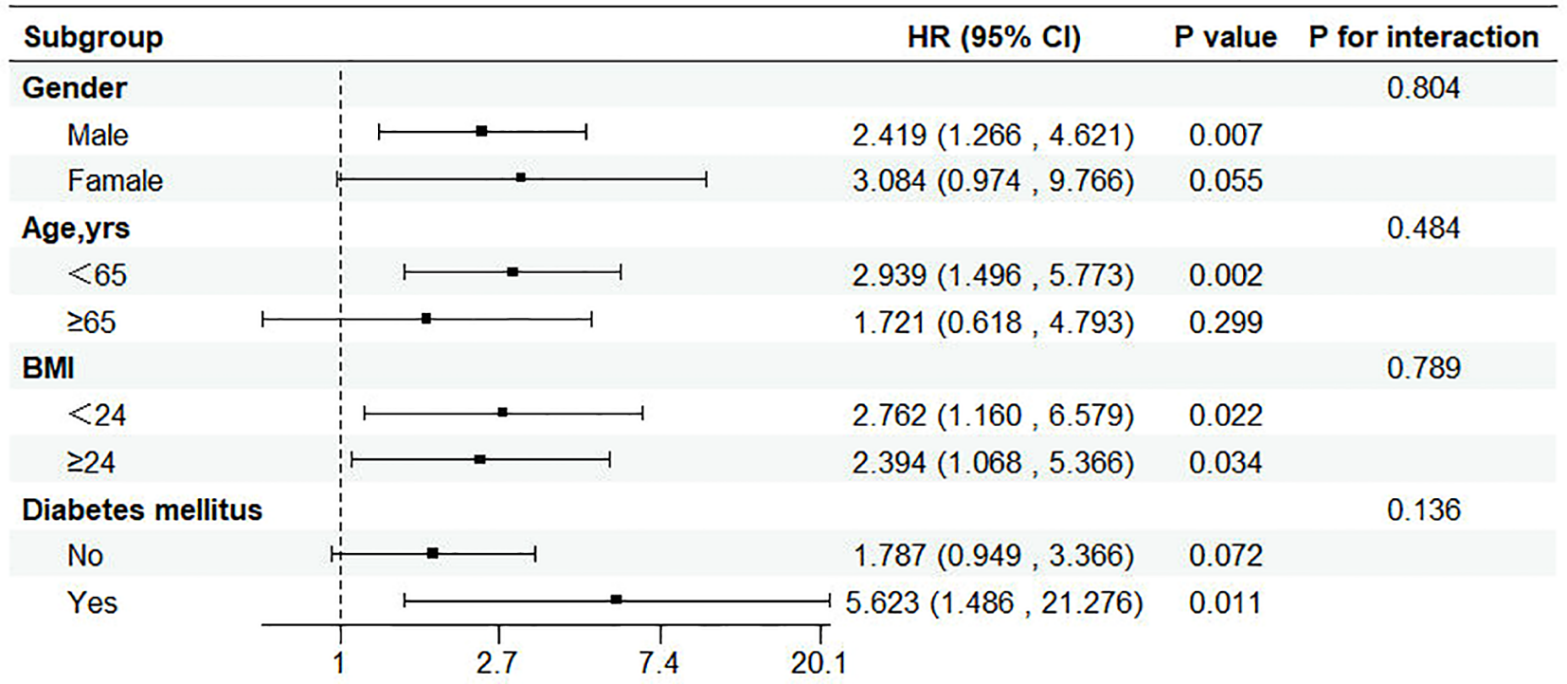
Subgroup analysis of the relationship between triglyceride glucose index and incident HF. CI, confidence interval; HR, hazard ratio.

Patients were classified according to age, sex, diabetes, BMI. No significant correlation with the risk of incident HF was found in the selected subgroup (P value > 0.05 for all interactions) (**Figure 5**).

## Discussion

Compared to the general population, patients undergoing maintenance hemodialysis (MHD) exhibit a significantly elevated incidence of heart failure (HF) (1, 19). MHD patients are subject to complex heart failure dependencies due to dialysis, like accelerated hemodynamic changes, sudden drops in blood pressure, dehydration, and electrolyte disturbances, uremic toxins, and heavy fluid loads (3, 4, 20). The TyG index, widely recognized as an effective marker for assessing insulin resistance (IR), has been gaining attention (8). The study explored the epidemiological relationship between the human ‘TyG index’ and the risk of this event. The findings conveyed a significant impact of the TyG index that could lead to the lethal heart event even though many other potential incident factors were taken into account. In the same line, a clear dose-response was also witnessed between TyG index and HF induction. To our best knowledge, this research has recorded the first trial being done to construct the relation between TyG index and the incidence of heart failure in the end-stage renal disease.

The incidence of heart failure (HF) in hemodialysis patients remains at a high level (21, 22). A major challenge in this research field is how to predict the risk of heart failure, identify specific predictors of HF, and thereby provide effective interventions as early as possible. Numerous studies have already confirmed a significant association between the TyG index and the risk of heart failure. In recent research, with regard to predicting incident HF, especially with regard to the TyG index, it has been increasingly supported. For instance, a new research study which was based on two big cohort studies and a Mendelian randomization analysis showed that the participants with the topmost level of the TyG index were at a greater risk of developing HF as compared to the rest of the participants (13). The essence of Mendelian randomization studies pointed out a causal relationship between an increased TyG index value and a rising occurrence of heart failure. Besides, an investigation suggested that the highest quartile of the TyG index was associated with a greater HF risk accompanied by the higher possibility of unfavorable left ventricular restructuring and function (23). Together, this study provides the evidence that there is an increase in the incidence of HF with increasing TyG index, which has been shown in the earlier study as well.

Although the majority of previous research accordingly focused on the general healthcare population, there is a remarkable scarcity of information about the implications of the TyG index in HF cases among the MHD patients, a high-risk population. Xie et al. (9) observed that an increase in the TyG index was associated with an increased risk of major adverse cardiovascular events (MACE) in patients with end-stage renal disease and coronary artery disease. To a similar effect, Yan et al. (24) studied found that high values of the TyG index are correlated with the poor results of cardiovascular pathology in peritoneal dialysis patients. Based on this scientific background, this study aims to analyze the relationship between the TyG index and the risk of HF by studying the MHD population on a large scale. Through this research, we showed that the TyG index is an independent predictor of incident HF in hemodialysis patients and is associated with the risk of heart failure even after adjusting for multiple effectiveness-confounding factors. In line with this, the higher TyG index group was associated with a higher risk of incident HF in comparison with the lower TyG group. Interestingly, we noted that the TyG index in MHD patients was also indicative of the incidence of HF independently of other variables.

The potential mechanisms underlying the relationship between the TyG index and HF in MHD patients can be elucidated through IR. Firstly, IR can cause myocardial energy metabolism disorders. As the central organ of the circulatory system, the heart requires a substantial energy supply for its uninterrupted myocardial motion to maintain normal function. Compared to fatty acid oxidation, glucose oxidation is more efficient in terms of energy production. However, IR leads to a decreased sensitivity of target organs, such as the heart, to glucose, resulting in reduced glucose utilization. The high energy demand prompts myocardial cells to increase their utilization of lipids, which subsequently decreases the production of ATP from glycolysis in the heart (25). IR also impairs the AMPK/PGC-1α signaling pathway, resulting in mitochondrial dysfunction, impaired cellular ATP synthesis, and promoting cell apoptosis (26). Besides glucotoxicity and lipotoxicity, hyperinsulinemia induced by IR, activation of the renin-angiotensin system, and dysregulation of cytokines and oxidative stress are also important contributors to cardiac IR and impaired cardiac function (27, 28).

Clinically, the TyG index is influenced by multiple factors. Theoretically, Theoretically, it can be assumed that if blood glucose and triglyceride levels in the blood can alter whether they are raised or reduced through the management of the disease state or drug therapy, this may consequently affect the predictive ability of the TyG index. However, an intriguing phenomenon revealed by Sun Sun et al. (29) is that the TyG index demonstrates consistent validity in predicting the risk of major adverse cardiovascular events (MACE) following percutaneous coronary intervention (PCI), regardless of whether the patients have diabetes. Our study results aligns with this finding, as we also observed a positive correlation between the TyG index and the incidence of HF in different subgroups, without significant interaction effects. Furthermore, through receiver operating characteristic (ROC) curve analysis, we found that the TyG index has certain predictive value for the occurrence of HF in MHD patients, suggesting that it can be used as a risk stratification assessment tool to help clinicians identify high-risk populations in a timely manner and initiate interventions as early as possible.

Additionally, the restricted cubic spline (RCS) analysis in this study revealed a linear dose-response relationship between the TyG index and the risk of incident HF, consistent with previous research results. Notably, we observed a slight decrease in HF risk when the TyG index significantly increased to a certain level, a finding not reported in previous studies. Previous studies have observed a phenomenon called “reverse epidemiology” in patients with end-stage renal disease, where the correlation between serum triglyceride levels and all-cause and cardiovascular mortality gradually weakens in the later stages of CKD. Some studies suggest that these seemingly contradictory associations may be the result of malnutrition-inflammation complex syndrome (30). However, due to the limited sample size of this study, we were unable to conduct a more in-depth investigation of this phenomenon. Therefore, more specific studies are needed in the future to further explore the complex relationship between higher TyG index levels and HF.

In this study, we also observed that patients with a higher TyG index had a higher incidence of HF-related rehospitalization compared to those with a lower TyG index. However, through ROC curve analysis, we found that the TyG index did not have good predictive value for HF-related rehospitalization in MHD patients (P > 0.05). This finding is inconsistent with the study by Liu et al. (31), which indicated that the TyG index was one of the independent risk factors both for HF-related rehospitalization and cardiovascular death in non-dialysis CKD stage 3–5 patients. Interestingly, however, a prospective multicenter observational study (32) found that insulin resistance in absence of diabetes mellitus (DM) does not impact the prognosis of congestive HF. Therefore, the role of TyG, as a reliable surrogate indicator of insulin resistance, in the prognosis of HF patients, still requires further investigation.

Despite the achievements of our study, there are certain limitations. Firstly, this study only used the baseline TyG index for analysis. To enhance the reliability of the research findings, we may consider calculating the cumulative average of the TyG index, utilizing it as a proxy for long-term TyG levels for further analysis. Secondly, although we have adjusted for various potential confounding factors in detail, we cannot completely exclude the influence of other unknown confounding factors on the study results. Thirdly, due to limitations in current clinical data, we were unable to fully assess the specific impact of dialysis adequacy on HF events. In another study (32), they pointing out a preponderant role of hyperglycemia (i.e., T2D) rather than insulin resistance on cardiovascular impairment. Therefore, more in-depth research is still needed in the future to fully reveal the specific mechanism of action of the TyG index in the occurrence of HF in dialysis patients.

## Conclusion

This study found that individuals with a higher TyG index in MHD patients have a significantly increased risk of incident heart failure. As a reliable, easy-to-calculate, and low-cost surrogate marker of IR, the TyG index has broad application prospects in clinical practice. This simple index may therefore facilitate recognition of patients at elevated risk of incident HF, provide important references for clinical interventions.

## Data Availability

The raw data supporting the conclusions of this article will be made available by the authors, without undue reservation.

## References

[1] Roehm B Gulati G Weiner DE. Heart failure management in dialysis patients: Many treatment options with no clear evidence. Semin Dial. (2020) 33:198–208. doi: 10.1111/sdi.12878 32282987 PMC7597416

[2] Khan MS Ahmed A Greene SJ Fiuzat M Kittleson MM Butler J. Managing heart failure in patients on dialysis: state-of-the-art review. J Card Fail. (2023) 29:87–107. doi: 10.1016/j.cardfail.2022.09.013 36243339

[3] House AA Wanner C Sarnak MJ Piña IL McIntyre CW Komenda P. Heart failure in chronic kidney disease: conclusions from a Kidney Disease: Improving Global Outcomes (KDIGO) Controversies Conference. Kidney Int. (2019) 95:1304–17. doi: 10.1016/j.kint.2019.02.022 31053387

[4] Romero-González G Ravassa S González O Lorenzo I Rojas MA García-Trigo I García-Fernández N. Burden and challenges of heart failure in patients with chronic kidney disease. A call to action. Nefrologia (Engl Ed). (2020) 40:223–36. doi: 10.1016/j.nefro.2019.10.005 31901373

[5] Riehle C Abel ED. Insulin signaling and heart failure. Circ Res. (2016) 118:1151–69. doi: 10.1161/CIRCRESAHA.116.306206 PMC4833475 27034277

[6] Artunc F Schleicher E Weigert C Fritsche A Stefan N Häring HU. The impact of insulin resistance on the kidney and vasculature. Nat Rev Nephrol. (2016) 12:721–37. doi: 10.1038/nrneph.2016.145 27748389

[7] Simental-Mendía LE Rodríguez-Morán M Guerrero-Romero F. The product of fasting glucose and triglycerides as surrogate for identifying insulin resistance in apparently healthy subjects. Metab Syndr Relat Disord. (2008) 6:299–304. doi: 10.1089/met.2008.0034 19067533

[8] Gastaldelli A. Measuring and estimating insulin resistance in clinical and research settings. Obes (Silver Spring). (2022) 30:1549–63. doi: 10.1002/oby.23503 PMC9542105 35894085

[9] Xie E Ye Z Wu Y Zhao X Li Y Shen N. The triglyceride-glucose index predicts 1-year major adverse cardiovascular events in end-stage renal disease patients with coronary artery disease. Cardiovasc Diabetol. (2023) 22:292. doi: 10.1186/s12933-023-02028-7 37891651 PMC10612201

[10] Ding H Zhu J Tian Y Xu L Song L Shi Y. Relationship between the triglyceride-glucose index and coronary artery calcification in asymptomatic, non-diabetic patients undergoing maintenance hemodialysis. Ren Fail. (2023) 45:2200849. doi: 10.1080/0886022X.2023.2200849 37133817 PMC10158539

[11] Xu L Wu M Chen S Yang Y Wang Y Wu S. Triglyceride-glucose index associates with incident heart failure: A cohort study. Diabetes Metab. (2022) 48:101365. doi: 10.1016/j.diabet.2022.101365 35660526

[12] Zheng H Chen G Wu K Wu W Huang Z Wang X. Relationship between cumulative exposure to triglyceride-glucose index and heart failure: a prospective cohort study. Cardiovasc Diabetol. (2023) 22:239. doi: 10.1186/s12933-023-01967-5 37667253 PMC10476374

[13] Li X Chan JSK Guan B Peng S Wu X Lu X. Triglyceride-glucose index and the risk of heart failure: Evidence from two large cohorts and a mendelian randomization analysis. Cardiovasc Diabetol. (2022) 21:229. doi: 10.1186/s12933-022-01658-7 36329456 PMC9635212

[14] Baek J He C Afshinnia F Michailidis G Pennathur S. Lipidomic approaches to dissect dysregulated lipid metabolism in kidney disease. Nat Rev Nephrol. (2022) 18:38–55. doi: 10.1038/s41581-021-00488-2 34616096 PMC9146017

[15] Legouis D Faivre A Cippà PE de Seigneux S. Renal gluconeogenesis: an underestimated role of the kidney in systemic glucose metabolism. Nephrol Dial Transplant. (2022) 37:1417–25. doi: 10.1093/ndt/gfaa302 33247734

[16] Rabbani N Thornalley PJ. Advanced glycation end products in the pathogenesis of chronic kidney disease. Kidney Int. (2018) 93:803–13. doi: 10.1016/j.kint.2017.11.034 29477239

[17] Soohoo M Moradi H Obi Y Kovesdy CP Kalantar-Zadeh K Streja E. Serum triglycerides and mortality risk across stages of chronic kidney disease in 2 million U.S. veterans. J Clin Lipidol. (2019) 13:744–753.e15. doi: 10.1016/j.jacl.2019.08.001 31562050

[18] McDonagh TA Metra M Adamo M Gardner RS Baumbach A Böhm M. 2021 ESC Guidelines for the diagnosis and treatment of acute and chronic heart failure [published correction appears in Eur Heart J. 2021 Dec 21;42(48):4901. doi: 10.1093/eurheartj/ehab670. Eur Heart J. (2021) 42:3599–726. doi: 10.1093/eurheartj/ehab368 34447992

[19] Cozzolino M Mangano M Stucchi A Ciceri P Conte F Galassi A. Cardiovascular disease in dialysis patients. Nephrol Dial Transplant. (2018) 33:iii28–34. doi: 10.1093/ndt/gfy174 PMC6168816 30281132

[20] Lai AC Bienstock SW Sharma R Skorecki K Beerkens F Samtani R. A personalized approach to chronic kidney disease and cardiovascular disease: JACC review topic of the week. J Am Coll Cardiol. (2021) 77:1470–9. doi: 10.1016/j.jacc.2021.01.028 33736830

[21] Murphy SP Ibrahim NE Januzzi JL Jr. Heart Failure With Reduced Ejection Fraction: A Review [published correction appears in JAMA. 2020 Nov 24;324(20):2107. doi: 10.1001/jama.2020.21736. JAMA. (2020) 324:488–504. doi: 10.1001/jama.2020.10262 32749493

[22] Wang Y Cao X Yu J Zhang Y Li X Chen X. Association of N-terminal pro-brain natriuretic peptide with volume status and cardiac function in hemodialysis patients. Front Cardiovasc Med. (2021) 8:646402. doi: 10.3389/fcvm.2021.646402 33693039 PMC7937607

[23] Huang R Lin Y Ye X Zhong X Xie P Li M. Triglyceride-glucose index in the development of heart failure and left ventricular dysfunction: analysis of the ARIC study. Eur J Prev Cardiol. (2022) 29:1531–41. doi: 10.1093/eurjpc/zwac058 35512245

[24] Yan Z Yu D Cai Y Shang J Qin R Xiao J. Triglyceride glucose index predicting cardiovascular mortality in Chinese initiating peritoneal dialysis: A cohort study. Kidney Blood Press Res. (2019) 44:669–78. doi: 10.1159/000500979 31315123

[25] Lopaschuk GD Karwi QG Tian R Wende AR Abel ED. Cardiac energy metabolism in heart failure. Circ Res. (2021) 128:1487–513. doi: 10.1161/CIRCRESAHA.121.318241 PMC8136750 33983836

[26] Jeong S Lee JH. The verification of the reliability of a triglyceride-glucose index and its availability as an advanced tool. Metabolomics. (2021) 17:97. doi: 10.1007/s11306-021-01837-9 34724122

[27] Erqou S Adler AI Challa AA Fonarow GC Echouffo-Tcheugui JB. Insulin resistance and incident heart failure: a meta-analysis. Eur J Heart Fail. (2022) 24:1139–41. doi: 10.1002/ejhf.2531 PMC9262840 35502564

[28] Hattori Y. Insulin resistance and heart failure during treatment with sodium glucose cotransporter 2 inhibitors: proposed role of ketone utilization. Heart Fail Rev. (2020) 25:403–8. doi: 10.1007/s10741-020-09921-3 31960270

[29] Sun C Hu L Li X Zhang X Chen J Li D. Triglyceride-glucose index’s link to cardiovascular outcomes post-percutaneous coronary intervention in China: a meta-analysis. ESC Heart Fail. (2024) 11:1317–28. doi: 10.1002/ehf2.14679 PMC11098636 38246749

[30] Soohoo M Streja E Hsiung JT Kovesdy CP Kalantar-Zadeh K Arah OA. Cohort study and bias analysis of the obesity paradox across stages of chronic kidney disease. J Ren Nutr. (2022) 32:529–36. doi: 10.1053/j.jrn.2021.10.007 PMC10032545 34861399

[31] Liu S Chen X Guo Q Dou J Huang J Jia L. Triglyceride glucose index: a significant prognostic marker of heart failure in patients with chronic kidney disease. Ren Fail. (2024) 46:2432547. doi: 10.1080/0886022X.2024.2432547 39604204 PMC11610345

[32] Salzano A D’Assante R Iacoviello M Triggiani V Rengo G Cacciatore F. Progressive right ventricular dysfunction and exercise impairment in patients with heart failure and diabetes mellitus: insights from the T.O.S.CA. Registry Cardiovasc Diabetol. (2022) 21:108. doi: 10.1186/s12933-022-01543-3 35710369 PMC9204878

