# Supplementary material for: Associations between the triglyceride–glucose index and the risk of heart failure in patients undergoing maintenance hemodialysis: a retrospective cohort study
Source: Front Endocrinol (Lausanne). 2025 Apr 3;16:1544591. doi: 10.3389/fendo.2025.1544591 (PMC12003116; doi:10.3389/fendo.2025.1544591)
Supplement: Supplementary file 1 [file DataSheet1.docx]

**supplementary matrial**

**SUPPLEMENTAL TABLE**

Supplementary Table 1. Comparison of baseline clinical characteristics between the two groups (80 patients with baseline echocardiographic data).

| Characteristic | TyG index＜8.60 | TyG index≥8.60 | t/z/χ² | *P* |
| --- | --- | --- | --- | --- |
| Glucose, mmol/L | 4.85（4.42,5.22） | 5.52（4.85,6.12） | -3.293 | 0.001 |
| TG, mmol/L | 0.95±0.28 | 1.97±0.59 | -9.436 | 0 |
| HF | 4（8.9%） | 12（34.3%） | 7.937 | 0.005 |
| Male（%） | 34（75.6%） | 27（77.1%） | 0.027 | 0.869 |
| Age, years | 52.77±13.12 | 49.37±13.88 | 1.254 | 0.214 |
| Duration of dialysis，mo | 33.00（19.00,62.50） | 25.00（12.00,74.00） | -0.922 | 0.357 |
| BMI | 23.66（20.49,25.44） | 24.21（21.01,26.67） | -0.407 | 0.684 |
| Heart rate，beats/min | 78.00（71.50,83.00） | 78.00（74.00,83.00） | -0.691 | 0.49 |
| SBP, mmHg | 149.36±18.64 | 150.54±26.09 | -0.203 | 0.84 |
| DBP, mmHg | 89.50（80.00,100.00） | 87.00（79.00,105.00） | -0.539 | 0.59 |
| Medical history , No. (%) |  |  |  |  |
| Hypertension | 34（75.6%） | 23（65.7%） | 0.931 | 0.335 |
| Diabetes mellitus | 7（15.6%） | 11（31.4%） | 2.845 | 0.092 |
| Stroke | 4（8.9%） | 4（11.4%） | 0 | 1 |
| Diabetic nephropathy | 5（11.1%） | 9（25.7%） | 2.908 | 0.088 |
| Discharge medications, No. (%) |  |  |  |  |
| Insulin therapy | 4（8.9%） | 5（14.3%） | 0.161 | 0.688 |
| ACE inhibitor or ARB | 12（26.7%） | 10（28.6%） | 0.036 | 0.85 |
| Beta-blocker | 14（31.1%） | 16（45.7%） | 1.791 | 0.181 |
| Erythropoietin | 27（60%） | 21（60%） | 0 | 1 |
| vitamin D | 6（13.3%） | 10（28.6%） | 2.857 | 0.091 |
| Hemoglobin, g/L | 85.00（75.50,91.50） | 84.00（71.00,100.00） | -0.058 | 0.954 |
| TC, mmol/L | 3.76±0.90 | 4.54±1.19 | -3.426 | 0.001 |
| HDL-C, mmol/L | 1.14±0.35 | 0.91±0.22 | 3.753 | 0 |
| LDL-C, mmol/L | 2.15（1.75,2.57） | 2.24（2.02,2.88） | -1.616 | 0.106 |
| Albumin，g/l | 40.03±5.36 | 37.91±6.75 | 1.556 | 0.124 |
| Ca (mmol/L) | 2.06（1.88,2.15） | 1.93（1.81,2.07） | -1.965 | 0.049 |
| P (mmol/L) | 1.96（1.65,2.34） | 2.08（1.67,2.47） | -0.708 | 0.479 |
| Serum urea，mmol/l | 32.93（28.58,41.99） | 38.53（28.81,44.40） | -1.45 | 0.147 |
| Serum creatinine, mg/dl | 792.00（682.50,1036.00） | 903.00（693.00,1172.00） | -0.8 | 0.424 |
| NT-pro BNP，pg/ml | 1672（779,3438.5） | 1134（731.6,524.8） | -0.31 | 0.756 |
| CK-MB，ng/ml | 2.02（0.91,3.36） | 2.3（1.48,4.30） | -1.15 | 0.25 |
| hs-TnT，ng/l | 41.50（22.68，59.50） | 52.30（31.34,99.30） | -1.761 | 0.078 |
| LVEF | 65.00（61.00,66.50） | 62.00（58.00,64.00） | -2.925 | 0.003 |
| LAD，mm | 36.00（33.00,41.00） | 38.00（34.00,41.00） | -0.617 | 0.537 |
| IVST，mm | 9.00（8.50,10.00） | 10.00（9.00,10.00） | -1.301 | 0.193 |
| LVEDD，mm | 49.00（46.00,50.00） | 49.00（47.00,54.00） | -1.495 | 0.135 |
| LVPWT，mm | 9.00（9.00,10.00） | 9.00（9.00,10.00） | -1.222 | 0.222 |
| LVMI，g/m^2^ | 99.15（88.59,116.12） | 105.27（87.89,123.93） | -1.043 | 0.297 |
| TRV，m/s | 2.70（2.50,2.88） | 2.63（2.40,2.80） | -1.126 | 0.26 |
| HF-related rehospitalization | 2（4.4%） | 7（20.0%） | 3.341 | 0.068 |
| All-cause death | 2（4.4%） | 0 |  | 0.502 |

Abbreviations: BMI Body mass index; ACE, angiotensin-converting enzyme; HF, heart failure; ARB, angiotensin receptor blocker; DBP, diastolic blood pressure; HDL-C, high-density lipoprotein cholesterol; LDL-C, low-density lipoprotein cholesterol; SBP, systolic blood pressure; TC, total cholesterol; TG, triglycerides; TyG, triglyceride-glucose; *Ca* ,Calcium; *P,* Phosphorus; NT-pro BNP, N-terminal pro-brain natriuretic peptide; hs-cTnT, high sensitivity-cardiac troponin T; CK-MB, creatine kinase-MB; LVEF, left ventricular ejection fraction; LAD, left atrial diameter; IVST, interventricular septum thickness; LVEDD, left ventricular end-diastolic diameter; LVPWT, left ventricular posterior wall thickness; LVMI, left ventricular mass index; TRV, Tricuspid Regurgitant Jet Velocity.

Supplementary Table 2. Univariate Cox regression model and Multivariate Cox regression model for the correlation between triglycerides-glucose index and the risk of incident heart failure (80 patients with baseline echocardiographic data). a Adjusted for covariates that were statistically significant in the univariate Cox regression model, including Beta-blocker, TG, TyG index, Duration of dialysis, LVEF. Clinically relevant risk factors were also adjusted in multivariate model, including age, male, BMI, Diabetes mellitus.

| Characteristic | Univariate analysis | | | Multivariate analysis | | |
| --- | --- | --- | --- | --- | --- | --- |
|  | HR | 95%CI | *P* | HR | 95%CI | *P* ^a^ |
| TyG index | 4.558 | 1.461-14.217 | 0.009 | 7.862 | 1.026-60.252 | 0.047 |
| Male | 0.923 | 0.296-2.873 | 0.889 | 2.414 | 0.618-9.427 | 0.205 |
| Age | 0.981 | 0.945-1.018 | 0.301 | 0.975 | 0.934-1.018 | 0.252 |
| BMI | 1.039 | 0.940-1.148 | 0.459 | 1.067 | 0.937-1.216 | 0.325 |
| Duration of dialysis | 0.974 | 0.950-0.998 | 0.032 | 0.969 | 0.941-0.998 | 0.035 |
| Diabetes mellitus | 1.665 | 0.527-5.261 | 0.385 | 1.02 | 0.269-3.876 | 0.976 |
| Beta-blocker | 5.247 | 1.670-16.490 | 0.005 | 5.034 | 1.308-19.375 | 0.019 |
| HDL-C | 0.245 | 0.051-1.180 | 0.079 |  |  |  |
| TG | 2.32 | 1.177-4.570 | 0.015 | 0.33 | 0.075-1.444 | 0.141 |
| LVEF | 0.901 | 0.833-0.974 | 0.008 | 0.941 | 0.863-1.026 | 0.167 |

Supplementary Table 3. Univariate Cox regression model and Multivariate Cox regression model for the correlation between triglycerides-glucose index and the risk of HF-related rehospitalization. a Adjusted for covariates that were statistically significant in the univariate Cox regression model, including BMI, Stroke, TyG index, Duration of dialysis. Clinically relevant risk factors were also adjusted in multivariate model, including age, male, BMI, Diabetes mellitus.

| Characteristic | Univariate analysis | | | Multivariate analysis | | |
| --- | --- | --- | --- | --- | --- | --- |
|  | HR | 95%CI | *P* | HR | 95%CI | *P* ^a^ |
| TyG index | 2.278 | 0.996-5.207 | 0.051 | 2.752 | 1.162-6.517 | 0.021 |
| Age | 1.024 | 0.996-1.054 | 0.099 | 1.03 | 0.994-1.066 | 0.1 |
| Male | 0.552 | 0.222-1.370 | 0.2 | 0.636 | 0.250-1.618 | 0.342 |
| BMI | 1.094 | 1.017-1.177 | 0.016 | 1.127 | 1.025-1.240 | 0.013 |
| Duration of dialysis | 0.969 | 0.951-0.988 | 0.001 | 0.966 | 0.946-0.985 | 0.001 |
| DBP | 0.977 | 0.953-1.001 | 0.065 |  |  |  |
| Diabetes mellitus | 1.997 | 0.893-4.463 | 0.092 | 1.26 | 0.512-3.098 | 0.615 |
| Stroke | 2.708 | 1.018-7.204 | 0.046 | 1.405 | 0.472-4.189 | 0.541 |
| Diabetic nephropathy | 2.235 | 0.936-5.337 | 0.07 |  |  |  |
| HDL-C | 0.327 | 0.099-1.083 | 0.067 |  |  |  |

**SUPPLEMENTAL FIGURE**

Supplementary Figure 1. Kaplan–Meier estimated the incidence of HF events based on triglyceride-glucose index grouping (80 patients with baseline echocardiographic data).


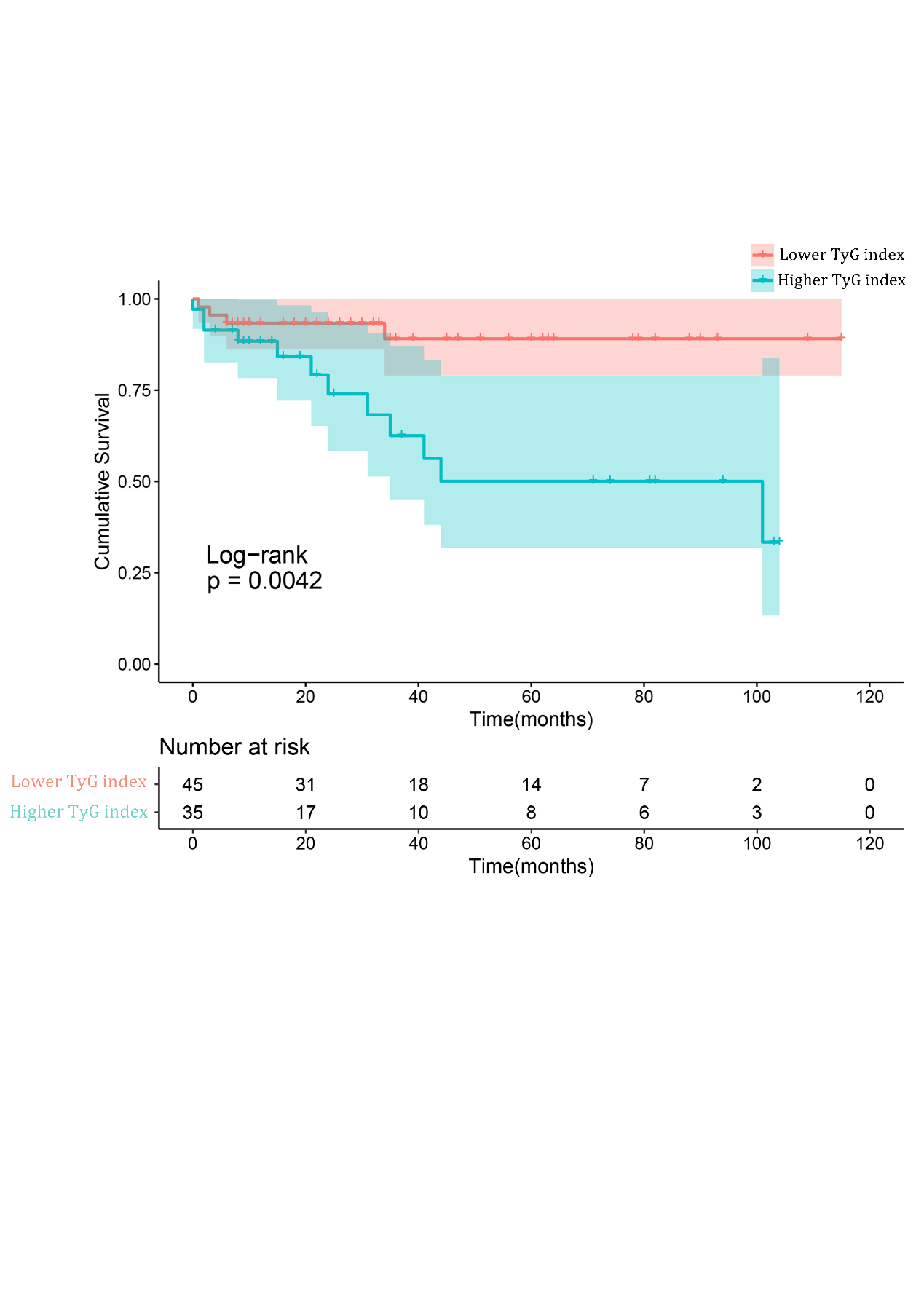


Supplementary Figure 2. ROC curve of TyG index in predicting incident heart failure in MHD patients (80 patients with baseline echocardiographic data).


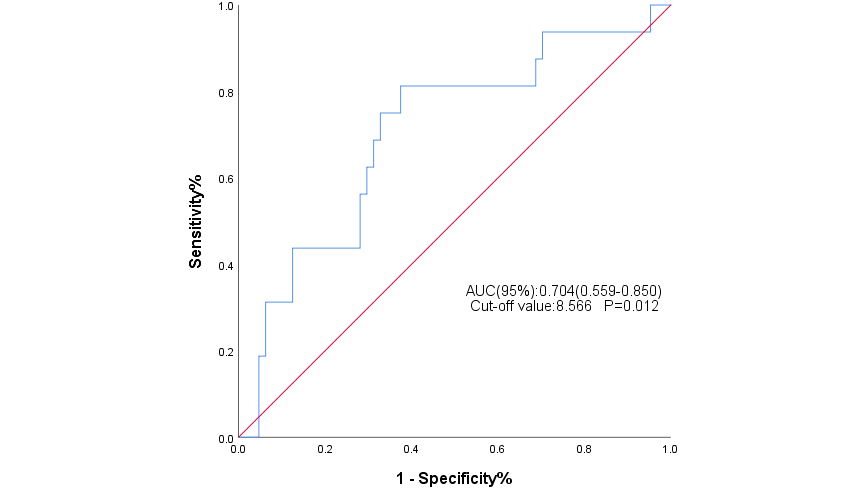


Supplementary Table 1. Comparison of baseline clinical characteristics between the two groups (80 patients with baseline echocardiographic data).

Supplementary Table 2. Univariate Cox regression model and Multivariate Cox regression model for the correlation between triglycerides-glucose index and the risk of incident heart failure (80 patients with baseline echocardiographic data) .

Supplementary Table 3. Univariate Cox regression model and Multivariate Cox regression model for the correlation between triglycerides-glucose index and the risk of HF-related rehospitalization.

Supplementary Figure 1. Kaplan–Meier estimated the incidence of HF events based on triglyceride-glucose index grouping (80 patients with baseline echocardiographic data).

Supplementary Figure 2. ROC curve of TyG index in predicting incident heart failure in MHD patients (80 patients with baseline echocardiographic data).
